# A Sparsity-Promoted Method Based on Majorization-Minimization for Weak Fault Feature Enhancement

**DOI:** 10.3390/s18041003

**Published:** 2018-03-28

**Authors:** Bangyue Ren, Yansong Hao, Huaqing Wang, Liuyang Song, Gang Tang, Hongfang Yuan

**Affiliations:** 1 College of Mechanical and Electrical Engineering, Beijing University of Chemical Technology, Beijing 100029, China; 2016200697@mail.buct.edu.cn (B.R.); 2017400138@mail.buct.edu.cn (Y.H.); 2018730001@mail.buct.edu.cn (L.S.); tanggang@mail.buct.edu.cn (G.T.); 2 Graduate School of Environmental Science and Technology, Mie University, 1577 Kurimamachiya-cho, Tsu, Mie 514-8507, Japan; 3 College of Information Science and Technology, Beijing University of Chemical Technology, Beijing 100029, China; yuanhf@mail.buct.edu.cn

**Keywords:** rotating machinery, sparse representation, feature enhancing, Majorzation-Minimization, fault detection

## Abstract

Fault transient impulses induced by faulty components in rotating machinery usually contain substantial interference. Fault features are comparatively weak in the initial fault stage, which renders fault diagnosis more difficult. In this case, a sparse representation method based on the Majorzation-Minimization (MM) algorithm is proposed to enhance weak fault features and extract the features from strong background noise. However, the traditional MM algorithm suffers from two issues, which are the choice of sparse basis and complicated calculations. To address these challenges, a modified MM algorithm is proposed in which a sparse optimization objective function is designed firstly. Inspired by the Basis Pursuit (BP) model, the optimization function integrates an impulsive feature-preserving factor and a penalty function factor. Second, a modified Majorization iterative method is applied to address the convex optimization problem of the designed function. A series of sparse coefficients can be achieved through iterating, which only contain transient components. It is noteworthy that there is no need to select the sparse basis in the proposed iterative method because it is fixed as a unit matrix. Then the reconstruction step is omitted, which can significantly increase detection efficiency. Eventually, envelope analysis of the sparse coefficients is performed to extract weak fault features. Simulated and experimental signals including bearings and gearboxes are employed to validate the effectiveness of the proposed method. In addition, comparisons are made to prove that the proposed method outperforms the traditional MM algorithm in terms of detection results and efficiency.

## 1. Introduction

Rotating machinery is widely applied in industrial fields. However, it generally operates under tough working conditions and is, therefore, subjected to mechanical failure. Faults occurring in rotating machinery may result in the fatal breakdown of the entire machinery system and can even cause casualties [1]. Therefore, condition monitoring and fault diagnosis of the rotating machinery have extremely vital significance. This is important for guaranteeing the production efficiency and plant safety in modern enterprises [2].

Since considerable fault information is displayed via vibration signals, the vibration-based diagnostic technique has emerged as an effective approach for mechanical fault diagnosis [3]. However, in the initial fault stage, fault features are comparatively weak. They possess low-amplitude and are mixed with more noise so that the fault features may be completely obscured by strong background noise [4]. Therefore, an efficient method is needed to enhance weak fault features and reduce noise [5]. Proper analysis for transient detection from the weak fault signals has attracted substantial scholarly attention throughout the past decades. Various methods have been proposed to extract weak fault features such as wavelet transform, empirical mode decomposition (EMD), higher order spectrum, and energy difference spectrum. Wang et al. [6] developed a fault feature enhancement method based on ensemble empirical mode decomposition (EEMD) and tunable Q-factor wavelet transform, which could extract the weak fault features of roller bearings. Chen et al. [7] studied a fault feature extraction method based on over complete rational dilation discrete wavelet transform (ORDWT), which aims to reduce the noise of fault signals and extract different types of fault features. The achieved results demonstrated that the ORDWT-based technique successfully identified the incipient fault features of gearbox. Tian et al. [8] proposed a novel modulation signal bispectrum (MSB) based robust detector for bearing fault detection, which allows effective suppression of both stationary random noise and discrete aperiodic noise and it can also enhance the modulation effects of a bearing fault and can be used to provide optimal frequency bands for fault detection. Zhang et al. [9] proposed a feature extraction method based on variational mode decomposition (VMD) and singular value energy difference spectrum in which the effectiveness is verified through wind turbine bearing fault experiments. Despite their relative successes, some drawbacks of existing methods persist. For instance, the decomposing thresholds of wavelet transform are not adaptive, but the method is easily influenced by multiple factors and the modal aliasing is always occurred based on EMD, which exacerbates the difficulty of feature extraction.

Given these weaknesses, an emerging method called sparse representation brought a new insight toward extracting the weak fault features [10]. There are several applications of sparse representation such as signal compression [11], de-noising [12], and weak fault feature extraction [13]. Sparse representation is employed to describe signals as linear combinations of a few atoms from a pre-specified dictionary. Research on sparse representation has thus far focused on two aspects [14]. First, scholars have examined the design of the sparse representation basis such as the orthogonal basis and over complete dictionary wherein representation coefficients can be computed as inner products of the signal and the pre-specified dictionary. Guo et al. [15] proposed a novel sparse representation method based on an online dictionary learning algorithm to satisfy real-time fault signal processing requirements. Second, pursuit methods have been applied toward solving the optimization problems such as the Basis Pursuit (BP) and Matching Pursuit (MP). In this case, the sparse coefficients of the most correlation between signals and atoms are calculated at each iteration, after which the sparsity vector can be obtained upon meeting the terminal iteration condition [16]. Fan et al. [17] proposed a sparse representation approach based on the traditional Majorzation-Minimization (MM) algorithm and by seeking to achieve fault signal de-noising and extract the weak fault features. Simulated studies and gearbox experiments both verified the effectiveness of the proposed method. However, the selection of sparse bases had a substantial impact on the fault diagnosis result. This method also contained more computations, which compromised efficiency [18].

In an effort to solve the aforementioned drawbacks and address the problem of weak fault feature extraction, an emerging method called Majorzation-Minimization algorithm brought a new insight to sparse representation. There are several applications of MM algorithm such as feature extracting, image processing, machine learning, and more. Jiang et al. [19] utilized MM algorithm to estimate the maximum likelihood of magnetic resonance images. Sun et al. [20] introduced the application of MM algorithm in signal processing, communication, and machine learning. In this paper, a novel sparse representation method based on the MM algorithm and BP algorithm was investigated to extract fault features. Essentially, the core idea of the MM algorithm is to utilize a series of simpler convex optimization problems to replace the original objective function [21]. However, the traditional MM algorithm must initially select an optimal sparse basis, which has a great impact on fault diagnosis results. Then a constrained optimization algorithm with the obtained special basis is incorporated to derive a series of sparse coefficients, which increases the computation and associated burden of fault diagnosis.

**Figure 1:**
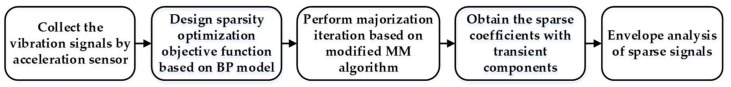
Flowchart of the proposed method.

In this study, a modified MM algorithm is investigated that does not depend on the choice of sparse basis and remarkably reduces the computation. The simple flowchart of the proposed method is shown in Figure 1 and specific steps and flowchart are described in Section 3. First, vibration signals are collected by acceleration sensors. Second, the sparse optimization objective function is designed based on the Basis Pursuit (BP) model, which integrates the impulsive feature-preserving factor and penalty function factor. The feature-preserving factor measures signal sparse representation error while the penalty function factor measures the sparsity of the fault signals. Third, a non-quadratic Majorization iterative method based on the modified MM algorithm is applied to address the convex optimization problem of the designed function. It is noteworthy that there is no need to select the sparse basis in the modified iterative method because it is fixed as a unit matrix. Signal reconstruction is also omitted in the modified MM algorithm, which significantly increases detection efficiency. With the aid of the proposed method, a series of sparse coefficients can be obtained in which the background noise can be reduced and the weak fault features can be highlighted. Finally, envelope analysis of sparse coefficients is conducted to extract the weak fault features. According to the proposed method, the period of transient impulses can be effectively identified and the beneficial characteristic frequencies can be extracted from the envelope spectrum of sparse signal.

The remaining parts of this paper are organized as follows. Section 2 describes the basic theory behind the BP algorithm and the Majorization iterative algorithm based on the MM method. The weak fault feature extraction strategy is outlined in Section 3. Simulated and experimental data, which consists of the roller bearing and gearbox, are presented in Section 4 along with a comparison method based on the traditional MM algorithm. Final conclusions are drawn in Section 5.

## 2. Basic Theory

Considering the problem of weak fault feature extraction, sparse representation is often applied to enhance the weak fault features and reduce noise. Consequently, the BP algorithm, which ensures that sparse signals contain adequate faulty information, is introduced in Section 2.1. The MM algorithm, which is capable of solving the convex optimization problem, is presented in Section 2.2.

### 2.1. Basis Pursuit Algorithm

This is fully obvious that signals induced by faulty mechanical components often mix with much noise. Therefore, the BP algorithm is applied to capture the useful fault characteristic *x* from the signal with noise *y* [22], which is expressed in Equation (1) below.

(1) y(t)=x(t)+n(t)

where *y*(*t*) is the sampled signal captured from the sensors, *x*(*t*) represents the characteristic signal containing the fault components, and *n*(*t*) denotes noise.

Basic Pursuit algorithm is adopted to identify redundant atoms from the library of the signal sparse representation after which the weak transient components are extracted from the sampled signal. The resultant model can be represented as Equation (2).

(2) y(t)=Dα(t)+n(t)

where $D\in R^{N\times M}$ is a matrix called orthogonal basis that contains *M* atoms $d_{i}\in R^{N},\;i=1,2\ldots M$. $\alpha \in R^{M}$ denotes the sparse representation coefficient.

Assuming that the sparse matrix *D* and sampled signal *y* are fixed, it is keen for obtaining the succinct representation coefficient, which demonstrates that the majority of the atoms in the coefficient vector are close to zero. This means only a handful of atoms will contribute to approximate the sampled signal [1]. To ensure the signal can be effectively expressed in sparse representation, Equation (2) can be converted into an optimization problem, which is shown below.

(3) $$\min{\left\|\alpha \right\|}_{0}\;s.t.\;\left\|y-D\alpha \right\|\leq \varepsilon$$

where ${\left\|\alpha \right\|}_{0}$ represents the number of nonzero items in *x* and ε denotes the error threshold.

Strictly speaking, *l*_0_ norm is the best method to measure the sparsity. Nevertheless, it doesn’t belong to a continuous function and, in this case, the continuous derivative cannot be achieved. To solve this problem, Chen S., Donoho D. L. et al. [23] proposed that utilizing the *l*_1_ norm to measure the sparsity, which can be converted in the following equation.

(4) $$\min{\left\|\alpha \right\|}_{1}\;s.t.\;{\left\|y-D\alpha \right\|}_{2}\leq \varepsilon$$

With the constraint of *l*_1_ norm minimization, sparse representation is defined as a series of constrained extremum problems that can be solved by a linear programming approach. Therefore, the aforementioned optimization problems can be expressed as seen in Equation (5).

(5) $$\hat{\alpha }=\underset{\alpha }{\arg\min}\left\{F\left(\alpha \right)=\frac{1}{2}{\left\|y-D\alpha \right\|}_{2}^{2}+\lambda {\left\|\alpha \right\|}_{1}\right\}$$

where F(α) is the sparse representation function.

There are two parts in this function. The first part is the impulsive feature-preserving factor measuring the signal sparse representation error and the second part is the penalty function factor measuring the sparsity of fault signals. Two variables exist in this function where one is the penalty factor λ that will adjust the weight of the two parts in the objective function. It is generally proportional to the transients and noise components in the signal. Another one is the sparse matrix *D*, which directly affects the degree of sparsity. In order to reduce computational complexity, the sparse matrix is fixed as a unit matrix.

In light of this convex optimization problem, the difference between sampled signal *y* and characteristic signal *x* can be gradually decreased such as where the noise components are reduced by minimizing F(α). Since it is difficult to minimize the object function F(α) unless it is quadratic, a Majorization iterative method based on the MM algorithm is applied in this study.

### 2.2. Majorzation-Minimization Algorithm

As mentioned earlier, the minimization of the object function F(α) can be easily solved if the function is quadratic. Based on this core idea, a modified MM approach is studied in which the objective function F(α) can be replaced by a sequence of simpler minimization problems [24]. $H_{k}(\alpha )$ is defined as the replaced function and satisfies the properties as seen in Equation (6).

(6) $$\begin{array}{l} \forall \alpha ,H_{k}\left(\alpha \right)\geq F\left(\alpha \right)\\ H_{k}\left(\alpha_{k}\right)=F\left(\alpha_{k}\right) \end{array}$$

To minimize F(α), an iterative algorithm based on MM is presented and a series of the sparse coefficients $\left\{\alpha_{k},\;k=1,2,3\ldots \right\}$ can be obtained according to Equation (7).

(7) $$\alpha_{k+1}=\underset{\alpha }{\arg\min}H_{k}(\alpha )$$

where *k* denotes iterations.

**Figure 2:**
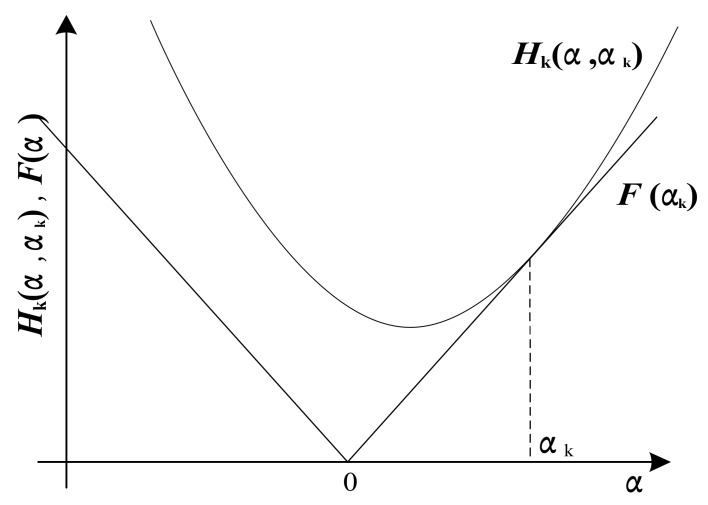
Sparse iterative algorithm based on MM.

Generally, the Majorization iterative algorithm consists of two sections. First, it consists of finding the initial value $\alpha_{0}$, meeting $F\left(\alpha_{0}\right)=H_{0}(\alpha_{0})$ and stipulating that the other values of F(α) are less than $H_{0}(\alpha )$, which is shown in Figure 2. Second, minimizing the objective function $H_{0}(\alpha )$ as the minimum value $\alpha_{1}$ so that $\alpha_{1}$ is a new iterative value making $F\left(\alpha_{1}\right)=H_{1}(\alpha )$, which is the same as the previous step. With an increase of iterations, the minimum value of the object function can be clearly obtained [24].

According to the sparse representation noted in Equation (5), two factors should be taken into account. One is the impulsive feature-preserving factor, which is more easily minimized due to its quadratic term. Therefore, it can be solved by running several steps of the conjugate gradient algorithm. The other one is the penalty function factor, mark it as ${\left\|\alpha \right\|}_{1}=\Psi \left(\alpha \right)$, term |α| as ϕ(α), and then $\Psi \left(\alpha \right)={\left\|\alpha \right\|}_{1}=\sum {}_{n=1}^{N}\left|\alpha \left(n\right)\right|=\sum {}_{n=1}^{N}\phi \left(\alpha \left(n\right)\right)$. ϕ(α) is an absolute value function, which imposes a challenge for minimization. According to MM algorithm, assume a quadratic function h(α) as seen in the following equation.

(8) $$h\left(\alpha \right)=A\alpha^{2}+B\alpha +C$$

where A, B, and C are constants.

The surrogate function h(α) should be the upper bound of ϕ(α) that agrees with ϕ(α) at $\alpha_{k}$, as shown in Figure 2. Therefore, conditions in Equation (6) are equivalent to $h(\alpha_{k})=\phi \left(\alpha_{k}\right)$, $h\prime (\alpha_{k})=\phi \prime \left(\alpha_{k}\right)$. In order to simplify the Majorization algorithm, consider a special form of h(α) that assumes an unknown parameter *B* = 0 [25]. Then, h(α) can be expressed as seen in the following equation.

(9) $$h\left(\alpha \right)=A\alpha^{2}+C$$

Next, solving for *A* and *C* makes $A=\left(\phi \prime \left(\alpha_{k}\right)/2\alpha_{k}\right),C=\phi \left(\alpha_{k}\right)-\left(\alpha_{k}/2\right)\phi \prime \left(\alpha_{k}\right)$. Then,

(10) $$h\left(\alpha \right)=\frac{\phi \prime \left(\alpha_{k}\right)}{2\alpha_{k}}\alpha^{2}+\phi \left(\alpha_{k}\right)-\frac{\alpha_{k}}{2}\phi \prime \left(\alpha_{k}\right)$$

According to the aforementioned algorithm, the conditions in Equation (6) are equivalent to the following equation.

(11) $$\begin{array}{l} \forall \alpha \;h_{k}\left(\alpha \right)\geq \phi \left(\alpha \right)\\ \frac{\phi \prime \left(\alpha_{k}\right)}{2\alpha_{k}}\alpha^{2}+\phi \left(\alpha_{k}\right)-\frac{\alpha_{k}}{2}\phi \prime \left(\alpha_{k}\right)\geq \Psi \left(\alpha \right) \end{array}$$

In attempting to simplify the concrete form of α, the above function will be converted into a matrix format.

(12) $$\frac{1}{2}\alpha^{*}\Lambda_{k}^{-1}\alpha +\frac{1}{2}{\left\|\alpha_{k}\right\|}_{1}\geq {\left\|\alpha \right\|}_{1}$$

where $\Lambda_{k}$ denotes the diagonal matrix with $\Lambda_{k}=diag\left(\left|\alpha_{k}\right|\right)$, $\Lambda_{k}^{-1}$ represents the inverse of $\Lambda_{k}$, and $\alpha^{*}$ denotes the complex conjugate transpose of α.

Incorporated with the impulsive feature-preserving factor, the majorization function $H_{k}(\alpha )$ can be expressed as Equation (13).

(13) $$H_{k}\left(\alpha \right)=\frac{1}{2}{\left\|y-D\alpha \right\|}_{2}^{2}+\lambda (\frac{1}{2}\alpha^{*}\Lambda_{k}^{-1}\alpha +\frac{1}{2}{\left\|\alpha_{k}\right\|}_{1})$$

According to the Majorization Iteration Algorithm, Equation (7) for α can be updated as seen below.

(14) $$\alpha_{k+1}=\underset{\alpha }{\arg\min}\left(\frac{1}{2}{\left\|y-D\alpha \right\|}_{2}^{2}+\frac{\lambda }{2}\alpha^{*}\Lambda_{k}^{-1}\alpha +\frac{\lambda }{2}{\left\|\alpha_{k}\right\|}_{1}\right)$$

The last part of this function $\frac{\lambda }{2}{\left\|\alpha_{k}\right\|}_{1}$ can be omitted because it is a fixed value. Therefore, a new updated equation can be simplified as seen in the equation below.

(15) $$\alpha_{k+1}=\underset{\alpha }{\arg\min}\left(\frac{1}{2}{\left\|y-D\alpha \right\|}_{2}^{2}+\frac{\lambda }{2}\alpha^{*}\Lambda_{k}^{-1}\alpha \right)$$

Equation (15) is thereby quadratic in α. With the aid of the traditional MM algorithm [17], the solution to this problem can be converted explicitly using linear algebra and leads to the following updated equation.

(16) $$\alpha_{k+1}={\left(D^{*}D+\lambda \Lambda_{k}^{-1}\right)}^{-1}D^{*}y$$

However, some issues remain and should be taken into account [17]. Due to the sparsity of α, when the atoms of α go to zero, those of $\Lambda_{k}^{-1}$ would go to infinity, which leads to incorrect results. To solve this issue, the updated equation is converted into Equation (17).

(17) $$\alpha_{k+1}=\frac{1}{\lambda }\Lambda_{k}\left[D^{*}y-D^{*}{\left(D\Lambda_{k}D^{*}+\lambda I\right)}^{-1}D\Lambda_{k}D^{*}y\right]$$

The other issue pertains to the selection of the optimal sparse matrix. This problem can be solved by correlation filtering. Afterward, the constrained optimization algorithm with the obtained special basis is incorporated to achieve a series of sparse coefficients.

By contrast, the sparse matrix in the modified MM algorithm doesn’t need to be selected because it is fixed as a unit matrix. The updated equation can be simplified using matrix inverse lemma, which will not lead to infinite values and erroneous results [26]. Furthermore, since the sparse matrix is a unit matrix, signal reconstruction can be omitted and thus $x_{k+1}=\alpha_{k+1}$. In summary, the modified MM algorithm can significantly reduce the computational complexity and markedly increase the detection efficiency. The simplified update equation is thus converted into the calculation below.

(18) $$x_{k+1}=y-D^{T}{\left(\frac{1}{\lambda }\Lambda_{k}+DD^{T}\right)}^{-1}Dy$$

By running the iterative procedure expressed in Equation (18), an optimal sparse signal $\hat{x}$ containing the fault transient components can eventually be obtained.

## 3. Weak Fault Feature Enhancing Strategy Using Sparsity-Promoted Method

As mentioned earlier, vibration signals captured from faulty mechanical components often mix with much noise by imposing significant obstacles to weak fault feature extraction. It is, therefore, reasonable to enhance weak features and reduce noise by sparse representation [4]. As such, a fault feature extraction strategy based on the BP algorithm and MM method is investigated in this paper.

**Figure 3:**
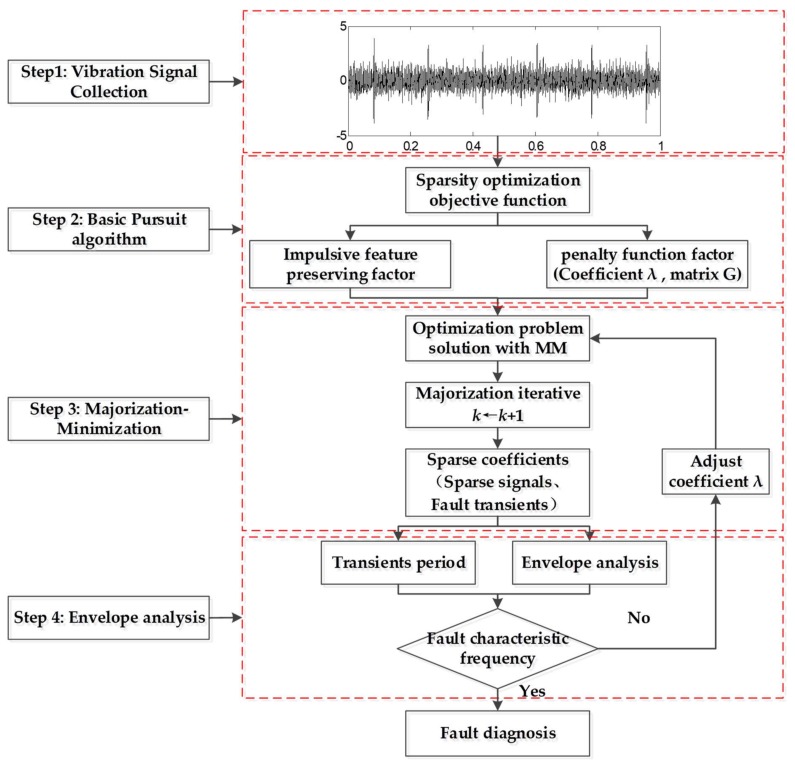
Feature enhancing algorithm framework based on MM.

The flowchart of the weak fault feature enhancing strategy is presented in Figure 3. The process is as follows: inspired by BP theory, a convex optimization objective function is designed that integrates the impulsive feature-preserving factor and penalty function factor. The feature-preserving factor measures the signal sparse representation error and the penalty function factor measures the sparsity of the fault signals. Essentially, this function reveals the differences between the fault signal without noise and the sampled signal. Then the Majorization iterative method based on the modified MM algorithm is applied to minimize this function. It is noteworthy that there is no need to select the sparse matrix G in the modified iterative method because it is fixed as a unit matrix. The penalty coefficient λ should be adjusted according to signal energy, which is generally proportional to the noise and fault impact components in the signal [27]. Through iteration, a series of sparse coefficients containing the transient components can be achieved. For the sparse matrix is the unit matrix, according to y=D∗x, the sparse signals are the same as sparse coefficients, which means signal reconstruction is omitted in the proposed method and inspired by sparse representation. The fault transients are reflected as the nonzero value of the sparse signal, which means it is the same as the sparse coefficients. Furthermore, the reciprocal of the transient period is consistent with the characteristic frequency. Finally, envelope analysis is employed to extract the fault features. Experiments on roller bearings and gearboxes were conducted in this study to verify the effectiveness of the proposed method. Comparison experiments show that the proposed algorithm outperforms the traditional MM method in terms of detection results and efficiency.

The details of this iterative procedure are summarized as seen below.

(1) Collect the vibration signals *y* using acceleration sensors,
(2) Construct the Majorization iterative function and initialize *k* = 1, sparse matrix *D*, penalty factor λ, and $\Lambda_{k}=diag\left(\left|\alpha_{k}\left(n\right)\right|\right)$,
(3) According to Equation (18), carry out the Majorization iterative steps and update the iterative number *k*←*k* + 1.
(4) Obtain sparse signal *x_k_*_+1_, which only contains the transient components.
(5) Perform envelope analysis and extract the fault characteristic frequency.

## 4. Application Cases

To demonstrate the effectiveness and applicability of the proposed method, experiments including simulated data, roller bearings, and gearboxes were conducted. In addition, comparisons were made to prove that the proposed method outperformed the sparse representation method based on the traditional MM algorithm.

### 4.1. Simulation Analysis

Vibration signals induced by bearing faults can be simulated as in the following equation.

(19) $$\begin{array}{c} y\left(t_{n}\right)=A\left(t_{n}\right)e^{-2\pi f_{n}\xi \left(t_{n}-T_{k}\right)}\times \sin\left(2\pi f_{n}\left(t_{n}-T_{k}\right)+\phi_{0}\right)+\;v\left(t\right),\\ T_{k}=T_{k-1}+\Delta T_{k},\;n,k=1,2,3,\ldots N-1 \end{array}$$

where $\left\{A\left(t_{n}\right),n=0,1,2,\ldots ,N-1\right\}$ denotes the amplitude of transient responses. The sampling frequency is *f_s_* = 10 kHz, the system resonance frequency is *f_n_* = 3 kHz, the initial phase angle is $\phi_{0}=5$ rad, and the relative damping ration is ξ = 0.1. *T_k_* represents the trigger time of the *k*th impulse and ∆*T_k_* = 0.01 is the time between the (*k* − 1)th and *k*th impulse in which fault characteristic frequency is 100 Hz. In order to simulate the background noise, Gaussian noise *v*(*t*) is added to the simulated signal and the SNR is −5 dB [28].

**Figure 4:**
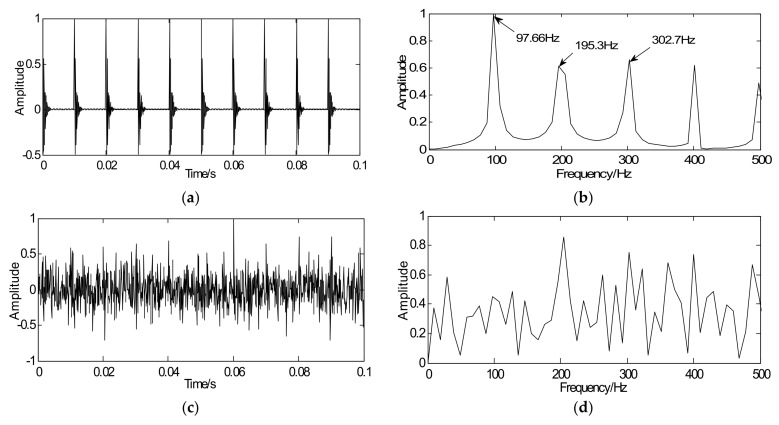
Simulated signals: (**a**) time-domain waveform of simulated signal, (**b**) envelope spectrum of simulated signal, (**c**) simulated signal with noise, and (**d**) envelope spectrum of signal with noise.

The signals in this paper are all normalized to guarantee the signal amplitude at the same magnitude. The waveform and the spectrum of the simulated signal are shown in Figure 4a,b, respectively, where the fault characteristic frequency can be obtained and is close to the theoretical frequency 100 Hz. To simulate the strong background noise, Figure 4c shows the waveform of the simulated signal with noise where the impact peak is unclear. Envelope analysis is selected as a comparison to validate whether the proposed algorithm can extract the weak fault features from the signal with noise. Fault frequencies of interest are submerged in the envelope spectrum, which is shown in Figure 4d.

**Figure 5:**
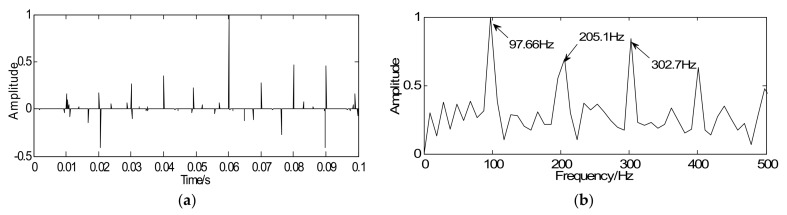
Detection results of simulated signals using the proposed method: (**a**) waveform of sparse signal and (**b**) envelope spectrum of the sparse signal.

As noted, the proposed method aims at enhancing the weak fault features and detecting features from the sparse signal. According to the proposed strategy, in the optimization iterative in Equation (18), the simulated signal with noise *y* is regarded as the initial iterative value, the sparse matrix G is marked as the unit matrix, the penalty coefficient λ=0.24, and iterations *k* = 20. Through iteration, a series of sparse coefficients only containing the transient components can be obtained, which is illustrated in Figure 5a. Figure 5b shows the envelope spectrum of the sparse signal where the defect frequency can be clearly extracted and the SNR of sparse signal is −0.649 dB. Compared with Figure 4c,d, the sparse signal demonstrates an excellent effect on transient detection. The noise can be well discarded and the fault characteristic frequencies can be clearly captured. Since the unit matrix is selected as the sparse basis, the reconstructed sparse signal is the same as the sparse coefficients. This means the signal reconstruction is omitted. Besides that, in order to verify the effectiveness of the proposed method for signals with different SNR, some supplementary experiments were also carried out and the following results were obtained: the SNR of simulated signals were −8 dB and −2, with the aid of the proposed method, the SNR of sparse signals could be got with −0.904 dB and −0.524 dB. Therefore, the proposed method, not only improves detection precision but also increases the detection efficiency.

### 4.2. Experimental Verification and Discussion

#### 4.2.1. Test Rig

**Figure 6:**
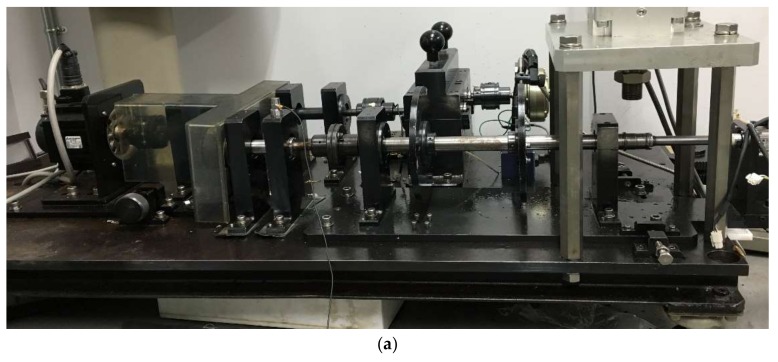

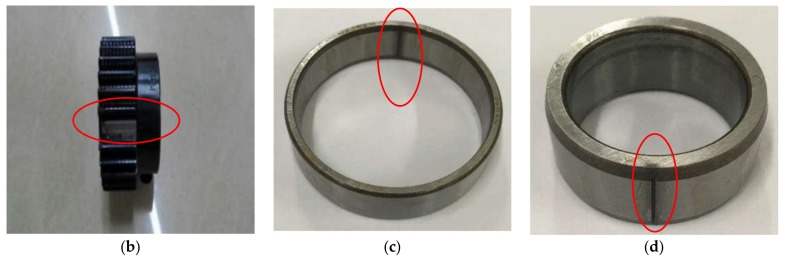
Experimental system, (**a**) experimental table, (**b**) gear broken-tooth fault, (**c**) bearing outer-race fault, and (**d**) bearing inner-race fault.

To verify the feasibility of the proposed method in practical engineering applications, several experiments were performed by testing set-ups with roller bearings and a gearbox. Figure 6a shows the testing set-up of roller bearings, which is comprised of a motor, roller bearings (NTN204), and acceleration sensors. Two accelerometers were mounted in opposite directions (horizontal and vertical) of the bearing housing. In addition, in this study, defective gearbox signals were analyzed. The testing set-up for the gearbox was equipped with a motor, a gearbox, loading equipment, and two accelerometers. The vibration signals of the defective gear were collected through two accelerometers mounted on the gearbox in vertical and horizontal directions. The fault gearbox was a two-stage gearbox that contained a driving shaft I (a driving gear), a middle shaft II (two gears: a driving gear and a driven gear), and a driven shaft III (a driven gear).

During the test, roller bearing faults occurred on the outer-race and inner-race, the gearbox fault occurred on the driven gear of the third speed, which is displayed in Figure 6b–d, and the red circles represent the location of faults. It is apparent that the inherent frequencies of roller bearing are generally distributed in the high-frequency band. To fully analyze the signal, a larger sampling frequency is selected to acquire more comprehensive information of the machinery conditions [29]. Therefore, the sampling frequency of bearing experiment was 100 kHz and the frequency of the gearbox experiment was 10 kHz. The roller bearing operated at a speed of 1300 rpm while the gearbox of the third speed operated at a speed of 340 rpm. The size of the flaw in the roller bearing was 0.05 mm × 0.3 mm (depth × width) in which the gear flaw was broken-tooth. The technical characteristics of the roller bearings and gearbox are displayed in Table 1 and Table 2. The corresponding fault characteristic frequencies of the bearing and gearbox are computed according to Equations (20) to (22) [30,31].

The fault characteristic frequency of bearing with outer race is shown in the equation below.

(20) $$f_{r}=\frac{N}{2}\left(1-\frac{d}{D}\cos\alpha \right)f_{0}$$

The fault characteristic frequency of bearing with inner race is shown in the equation below.

(21) $$f_{h}=\frac{N}{2}\left(1+\frac{d}{D}\cos\alpha \right)f_{0}$$

The fault characteristic frequency of gear with broken-tooth is shown in the equations below.

(22) $$\begin{array}{c} f_{m}=Z_{1}f_{1}=Z_{2}f_{2}\\ f_{z}=\frac{f_{m}}{Z} \end{array}$$

where *N* denotes the number of roller elements, *D* is the pitch diameter, *d* is the roller diameter, $f_{0},\;f_{1}$ and $f_{2}$ represent the rotating frequency of roller bearing, driving gear, and driven gear, respectively, α is the contact angle, $f_{r},\;f_{h},\;f_{z}$ represent the characteristic frequency of the outer race, inner race, and broken-tooth, respectively, $f_{m}$ is the gear meshing frequency, and $Z_{1},\;Z_{2}$ denote the number of gear teeth. Based on these parameters, the computed results of characteristic frequencies are listed in Table 3.

#### 4.2.2. Detection of the Bearing Fault in the Outer Race

**Figure 7:**
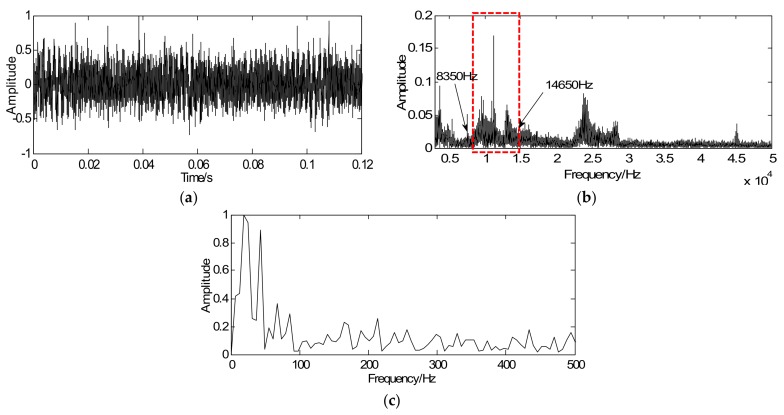
Bearing signals: (**a**) time-domain signal with outer-race fault, (**b**) spectrum of the time-domain signal, and (**c**) envelop spectrum of the time-domain signal.

To demonstrate the effectiveness of the proposed method for weak feature extraction, the proposed method is initially utilized to extract the weak fault features of roller bearing with a fault in the outer race. Figure 7a depicts the time-domain waveforms of the vibration signal in which the transients submerge in other noise components. Accordingly, it is difficult to detect fault transients as the transient cyclic period cannot be captured clearly. Envelope analysis is selected as a comparison to validate whether it is possible to extract the weak fault features from the vibration signal. Figure 7b shows the spectrum of Figure 7a in which the resonance bands can be selected as 8000–15,000 Hz and then calculate envelop spectrum, which is illustrated in Figure 7c. However, the fault frequencies of interest are too weak to extract in the envelope spectrum.

**Figure 8:**
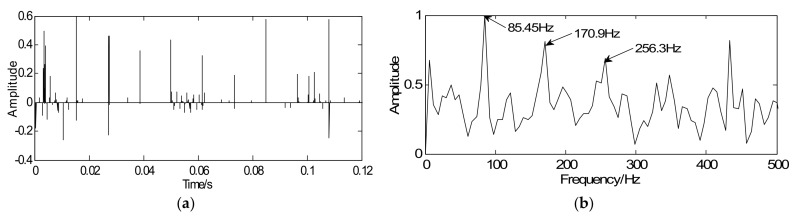
Detection results of outer-race fault using the proposed method: (**a)** waveform of sparse signal and (**b**) envelope spectrum of the sparse signal.

Based on the proposed method, a convex optimization objective function of the roller bearing is designed and inspired by BP theory. The sampled signal is regarded as the initial value *y* of the majorization iterative. Considering fault diagnosis efficiency, a unit matrix is chosen as the sparse orthogonal matrix *G*. Next, simplify the objective function and convert it into the modified iterative expression in which the penalty coefficient λ=0.32 and iterations *k* = 20. Via the above steps, the vibration signals can be sparsely represented. As shown in Figure 8a, the proposed method is effective in reducing noise and the signal only contains the fault impact peak. Figure 8b displays the envelope spectrum of the sparse signal where weak fault feature frequencies can be successfully extracted and it is consistent with the theoretical frequency (86.32 Hz). Compared Figure 7a with Figure 7c, the sparse signal exerts an excellent effect on transient detection. The transient cyclic period can be captured accurately and the fault characteristic frequencies can be clearly extracted. Since the unit matrix is selected as the sparse basis, the reconstructed sparse signal is the same as the sparse coefficients. In other words, signal reconstruction is omitted. Therefore, the proposed method improves detection precision along with detection efficiency.

#### 4.2.3. Detection of the Bearing Fault in the Inner Race

**Figure 9:**
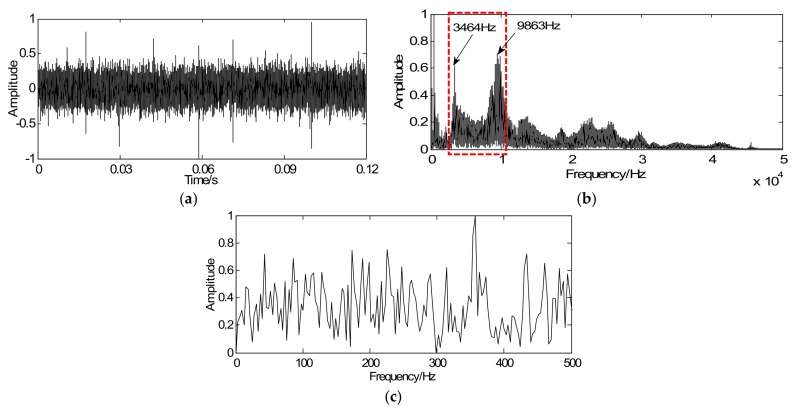
Bearing signals: (**a**) time-domain signal with inner-race fault, (**b**) spectrum of the time-domain signal, and (**c**) envelope spectrum of the time-domain signal.

The proposed method is then utilized to extract the weak fault features of the roller bearing with a fault in the inner race. Figure 9a shows the time-domain waveforms of the vibration signal in which most transients are submerged in other noise components. Envelope analysis is selected as a comparison to validate whether it can extract the weak fault features from the vibration signals. Figure 9b shows the spectrum of Figure 9a in which the resonance bands can be selected as 3000–10,000 Hz and then calculate envelop spectrum. However, as shown in Figure 9c, the fault frequency is hidden in other noise frequencies and the status of the roller bearing is difficult to identify.

**Figure 10:**
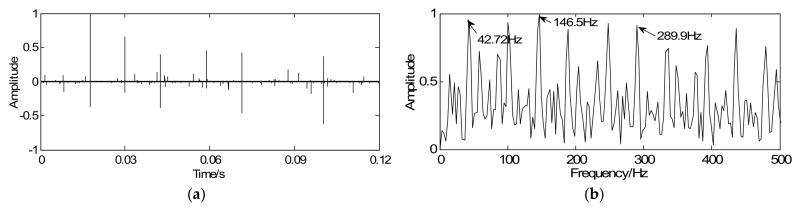
Detection results of inner-race fault using the proposed method: (**a**) waveform of sparse signal and (**b**) envelope spectrum of the sparse signal.

According to the proposed strategy, with the optimization iterative Equation (18), the vibration signal is regarded as the initial iterative value *y*, the sparse matrix *G* is marked as the unit matrix, the penalty coefficient λ=0.55, and iterations *k* = 20. Through iteration, the vibration signal can be sparsely represented and the weak fault feature can be clearly enhanced. As illustrated in Figure 10a, a series of sparse coefficients containing the transient components can be achieved. Figure 10b shows the envelope spectrum of the sparse signal in which the twice rotating frequency equaling 42.72 Hz and the defect frequency equaling 146.5 Hz can be obviously extracted. Due to the existence of the structural defects in testing set-up such as misalignment fault, the twice rotating frequency (42.72 Hz) can be clearly extracted in Figure 10b. Compared Figure 9a with Figure 9c, the sparse signal demonstrates an excellent effect on transient detection: the transient cyclic period can be well captured and the fault characteristic frequencies can be clearly extracted.

#### 4.2.4. Detection of the Gearbox Fault with Broken-Tooth

**Figure 11:**
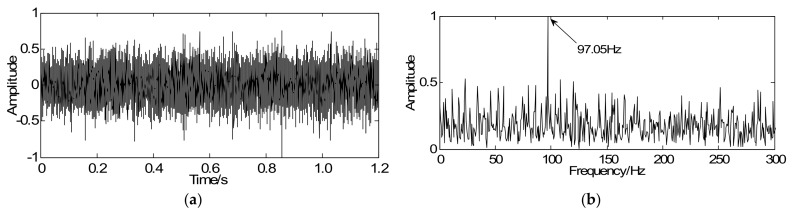
Gearbox signals: (**a**) time-domain signal with tooth-broken fault and (**b**) envelope spectrum of the time-domain signal.

To prove the commonality of this method, the proposed method is utilized to analyze the gearbox signal. The vibration signal caused by the gear with tooth-broken is shown in Figure 11a in which the transient period cannot be identified due to the existence of noise and other interfering components. As illustrated in Figure 11b, the meshing frequency can be extracted equal to 97.05 Hz and it is consistent with the theoretical frequency (97.13 Hz). Consequently, the malfunction appeared to occur on the second meshing, which may be the driving gear II or the driven gear III. However, it is impossible to diagnose which gear is faulty since the fault characteristic frequency is too weak to extract.

**Figure 12:**
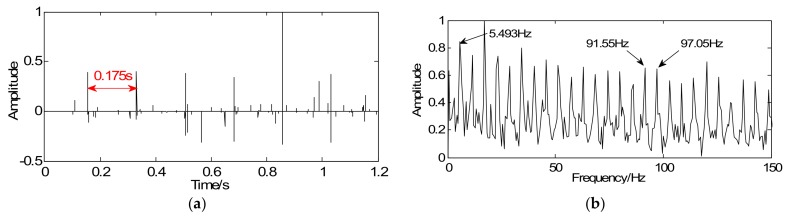
Detection results of tooth-broken fault using the proposed method: (**a**) waveform of sparse signal and (**b**) envelope spectrum of the sparse signal.

At a constant rotating speed, the localized fault in the gearbox tends to cause periodic shocks and thereby arouses periodic transients in vibration signals. Therefore, the gearbox fault can be diagnosed based on the transient period. However, due to the weak faulty, the fault transient period is obscured by strong background noise. Therefore, it is necessary to enhance the weak features. With the help of the proposed method, a convex optimization objective function (18) is designed. The sampled signal is regarded as the initial value *y* of the Majorization iterative. Considering fault diagnosis efficiency, a unit matrix is chosen as the sparse orthogonal matrix *G*. Next, simplify the objective function and convert it into the modified iterative expression, the penalty coefficient λ=2.7, and iterations *k* = 20. Via the above steps, the vibration signals can be sparsely represented as a series of periodic transients and the cyclic period $\Delta T=1/f_{z}=0.175\;s$ can be obtained in Figure 12a, which corresponds to the theoretical fault characteristic frequency (5.714 Hz). Figure 12b shows the envelope spectrum of the sparse signal in which the defect frequency, the meshing frequency, and its side band can be obviously extracted. Based on the mentioned analysis, the fault appears to occur on the driven gear III. Compared with the vibration signal in Figure 11a, it is disclosed that the sparse signal represents an excellent effect on transient detection as the transients’ cyclic period can be clearly captured as well as fault characteristic frequencies can be extracted obviously.

### 4.3. Comparison with the Traditional MM Algorithm

To further validate the superiority of the proposed method on detection results and efficiency, the same signals are processed using the traditional MM algorithm in which the optimal wavelet bases parameters of different research objects are firstly constructed by correlation filtering. Different wavelet bases may result in different detection results, which may lead to poor detection. Next the constrained optimization algorithm with obtained special basis is incorporated to achieve a series of sparse coefficients and the reconstructed signal can eventually be obtained through the sparse coefficients and special basis. In general, the traditional approach mainly involves correlation filtering, the constrained optimization algorithm, and signal reconstruction, which may increase the amount of calculation. By contrast, there is no need to select sparse basis in the modified MM algorithm or to reconstruct the signals for the sparse matrix is fixed as the unit matrix. Therefore, the modified MM algorithm can reduce computational complexity and boost detection efficiency considerably.

**Figure 13:**
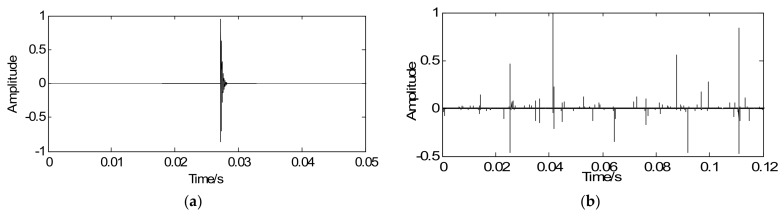

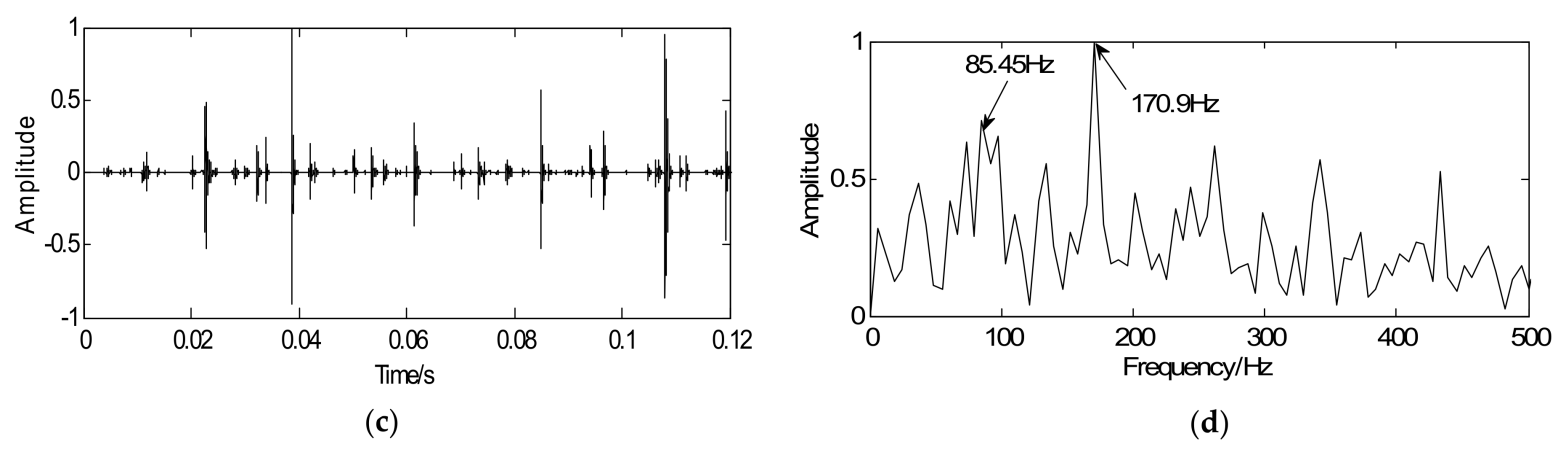
Detection results of outer-race fault signals using the traditional method: (**a**) Optimal wavelet basis, (**b**) sparse coefficients, (**c**) reconstructed signal, and (**d**) envelope spectrum of the reconstructed signal.

**Figure 14:**
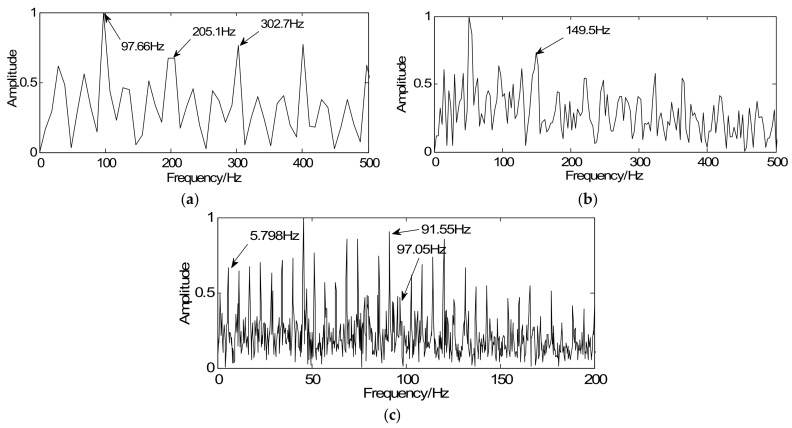
Detection results using the traditional method: (**a**) envelope spectrum of simulated sparse signal, (**b**) envelope spectrum of the inner-race sparse signal, and (**c**) envelope spectrum of the gearbox sparse signal.

The traditional method is firstly utilized to extract the weak fault features of the roller bearing with outer-race fault. The bearing signal is shown in Figure 7a and its spectrum is illustrated in Figure 7b. According to the method based on the traditional MM algorithm, the wavelet basis can be obtained via correlation filtering (see Figure 13a). Then the constrained optimization algorithm with obtained special basis is incorporated to achieve a series of sparse coefficients (see Figure 13b). Figure 13c illustrates the reconstructed signal where the noise can be reduced effectively, but the transient cyclic period cannot be clearly observed. Figure 13d displays the envelope spectrum of the reconstructed signal. The feature frequencies of interest are not clear. Then the traditional method is utilized to extract the weak fault features of the simulated fault, the roller bearing with inner-race fault and gearbox fault, which is shown in Figure 14a–c. Compared with the detection results using the proposed method, the modified method shows better transient detection as the fault characteristic frequencies can be extracted more obviously.

**Figure 15:**
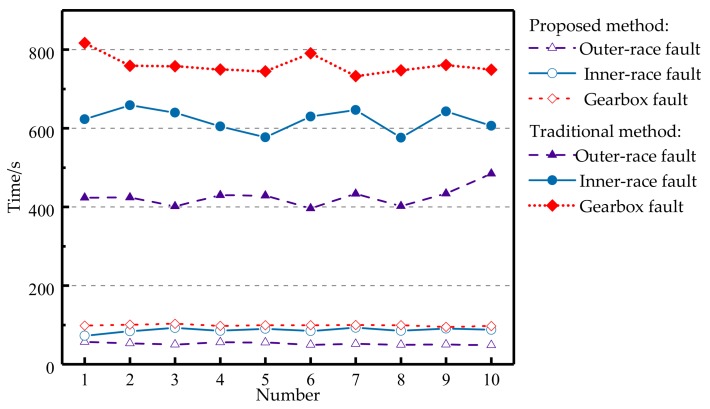
Detection time using two methods.

To substantiate the efficiency of these two methods, several comparative experiments were carried out to obtain the detection time. The average detection time of the simulated signal using the proposed method is 0.1865 s and the average detection time using the traditional method is 9.0883 s. Due to the fewer data points, there are fewer differences in fault detection time of the simulated signal. However, the detection time of bearing and gear fault signals is quite different actually. Figure 15 shows the detection time for 10 sets of experiments using the two methods. It can be seen that the detection time of the proposed method is far less than that of the traditional method. Therefore, the proposed method can greatly reduce computational complexity and significantly increase detection efficiency.

## 5. Conclusions

In this study, a sparsity-promoted method based on the BP and modified MM algorithm is proposed to extract weak fault features. The optimization objective function is initially designed to make the noise-polluted signal approximate to the transient impact, which means that fault feature enhancement can be converted into a convex optimization problem. Then the Majorization iterative method based on a modified MM algorithm is applied to solve the convex optimization problem. Through iteration, not only the strong background noise reduces but also a series of sparse coefficients only containing transient components can be obtained. To verify the validity of the proposed method, simulated and experimental signals of bearings and gearboxes are analyzed. The results indicate that the sparse signal represents an excellent effect on transient detection. The transient cyclic period can be well captured and the fault characteristic frequencies can be clearly extracted. Envelope analysis is selected as a comparison to validate whether it can extract the weak fault features from the vibration signal. It can be seen that the fault frequencies of interest are too weak to extract in the envelope spectrum, but they can be successfully extracted using the proposed method. In addition, comparisons using the traditional method demonstrate that the proposed method outperforms the traditional method in terms of detection results and efficiency.

## Figures and Tables

**Table 1:** Structure parameters of roller bearing.

Number of Rollers	External Diameter (mm)	Inner Diameter (mm)	Width (mm)
11	47	20	14

**Table 2:** Structure parameters of gearbox.

Research Object	Number of Teeth	Rotating Period (s)	Rotating Frequency (Hz)	Meshing Frequency (Hz)
Driving Gear I	80	3.448	0.29	23.15
Driven Gear II	19	0.824	1.214	
Driving Gear II	80	0.824	1.214	97.13
Driven Gear III	17	0.175	5.714	

**Table 3:** Fault characteristic frequencies.

Fault Category	Outer Race	Inner Race	Broken-Tooth
Fault characteristic frequency (Hz)	86.32	145.84	5.714
